# The remaining dentin thickness investigation of the attempt to remove broken instrument from mesiobuccal canals of maxillary first molars with virtual simulation technique

**DOI:** 10.1186/s12903-015-0075-x

**Published:** 2015-07-28

**Authors:** Qian Yang, Gary Shun-Pan Cheung, Ya Shen, Dingming Huang, Xuedong Zhou, Yuan Gao

**Affiliations:** Department of Operative Dentistry & Endodontics, State Key Laboratory of Oral Diseases, West China College & Hospital of Stomatology, Sichuan University, 14#, 3rd section of RenMin South Road, Chengdu, 610041 China; Area of Endodontics, Comprehensive Dental Care, Faculty of Dentistry, University of Hong Kong, Hong Kong, China; Division of Endodontics, Department of Oral Biological & Medical Sciences, Faculty of Dentistry, University of British Columbia, Vancouver, Canada

**Keywords:** Broken instrument, Virtual simulation, Periapical radiography, Remaining dentin thickness

## Abstract

**Background:**

To investigate differences in the estimated minimum remaining dentin thickness (RDT) between periapical radiographs using the paralleling and parallax technique, after simulated removal of broken instrument from the mesiobuccal (MB) canal of maxillary first molar in virtual simulation model. The 3D measurement was taken as the standard for comparison.

**Methods:**

Thirty-six maxillary first molars were scanned by micro-CT and reconstructed as 3-dimensional (3D) model. A virtual fragment of an instrument was created within the MB canal in software. Removal of the broken instrument was simulated in both the 3D and 2D dataset. Then, the models of all specimens were submitted to 2D and 3D measurements for the lowest (RDT) value in each. Differences in the values between the paralleling and parallax radiographic technique and the 3D-RDT value were analyzed with two-way Analysis of Variance. The Intra-class Correlation Coefficient (ICC) was used to assess consistency of the RDT measurements between the two periapical radiographic and techniques and 3D analysis.

**Results:**

There was significant difference between RDT value obtained from the paralleling technique and 3D-RDT. There were no differences between RDT obtained from parallax (angled) technique and 3D-RDT. The ICC of RDT values between paralleling technique and 3D measurement were lower than 0.75. ICC between angled radiographs and 3D technique was close to 0.75. The optimal horizontal angle for the parallax technique was about 21°.

**Conclusions:**

The virtual simulation technique can provide valuable insight into the benefit/risk analysis before removal of a broken instrument. Parallel radiographs overestimate the actual remain dentin thickness in mesiobuccal canals of maxillary first molars, whereas the parallel technique would give a closer estimate to the actual thickness at a projection angle of about 21°.

## Background

Root canal preparation is an essential stage of root canal treatment aiming to clean and shape the canals thoroughly. The introduction of rotary nickel-titanium (NiTi) endodontic instruments has improved the efficacy of the process compared with manual stainless steel files [1], as well as enhanced the success rate of treatment [2]. There is a concern about the separation of instrument [3], which has been reported to occur most often in the mesiobuccal canal of maxillary molars and mesial canal of mandibular molars, due to their canal curvature and complex anatomy [4]. The presence of a broken fragment would hinder the thoroughly cleaning and shaping of the root canal system, and may affect the long-term prognosis of treatment [5].

In considering the removal of broken instruments, the clinician needs to evaluate the risk and consider the possible complications. Excessive loss of dentin can increase the risk of lateral perforation or root fracture [6]. The remaining dentin thickness (RDT) is probably the most important factor affecting the decision of removing the fragment instrument, as that contributes to the resistance against root fracture [7, 8]. Typically, the RDT is estimated on periapical radiographs. According to Lim and Stock [8], 200 to 300 μm of dentin thickness should be present after preparation, to withstand the compaction forces during obturation to prevent perforation or fracture. If RDT falls below a certain value, it would be risky to attempt removal of the fragment. Instead, one may then attempt to bypass the broken instrument, or to clean/shape and fill the root canal up to the fragment [9]. Earlier studies usually sectioned the tooth to measure the canal wall thickness in cross section [9–11]. Such method is destructive, and the samples cannot be used for further studies or as their own control. Furthermore, it is not easy to compare the results with other reports, because of the variability of root canal anatomy. Recently, micro-computed tomography (micro-CT) and the technique of virtual simulation provide promising applications in endodontic research [12, 13]. Micro-CT is as a non-destructive method that has been used to investigate the three-dimensional (3D) morphologic features of roots and root canals. Tomographic images are digitally reconstructed in 3 dimensions [14]. Simulated 2-dimensional (2D) radiographs can be generated, based on micro-CT data by a direct ray casting technique in software, without taking a real radiograph [15–17]. Thus, one can measure and calculate the dentin thickness from 3D micro-CT data and the 2D simulated radiographs.

Although radiographs are widely used in clinical endodontics, they are not accurate for determining the actual root anatomy, because of distortion and presence of overlapping structures. In addition, film-based radiograph has the limitation of being two-dimensional projection of a three-dimensional object [18]. For instance, the zygomatic process typically overlaps the roots of maxillary first molar. So, some details about the root anatomy can be misinterpreted or lost, which hinder the visualization of the root canal anatomy and any concavities that may be present in the proximal root surface. This may compromise clinical judgment, especially when the decision to remove broken instrument is concerned. There are few reports on the evaluation and calculation of dentin thickness before the removal of broken instrument in maxillary first molars by radiographic means. The purpose of this study was to evaluate the remaining dentin thickness measurements based on paralleling and parallax (angle) radiographic image, versus 3D tomography, after the virtual removal of broken instruments from the mesiobuccal canal of maxillary first molars.

## Methods

Thirty-six maxillary first molars were selected from a collection of extracted human teeth from a Chinese population sample based on mature apices without visible apical resorption. After understanding and written consent was obtained from patients, the extracted teeth were collected by the West China Hospital of Stomatology for teaching and research. The present study was approved by the Ethics Committee of the West China Hospital of Stomatology, and the molars were selected from the teeth bank of the hospital. These teeth were ultrasonically cleaned and stored in thymol solution until use. The teeth were scanned by using a micro-CT system (microCT-50, Scanco Medical, Bassersdorf, Switzerland) with an isotropic voxel size of 30 μm. All scanned data were processed on an HP 6600 W workstation [Hewlett Packard, Palo Alto, CA] running Windows 7.

**Fig. 1:**
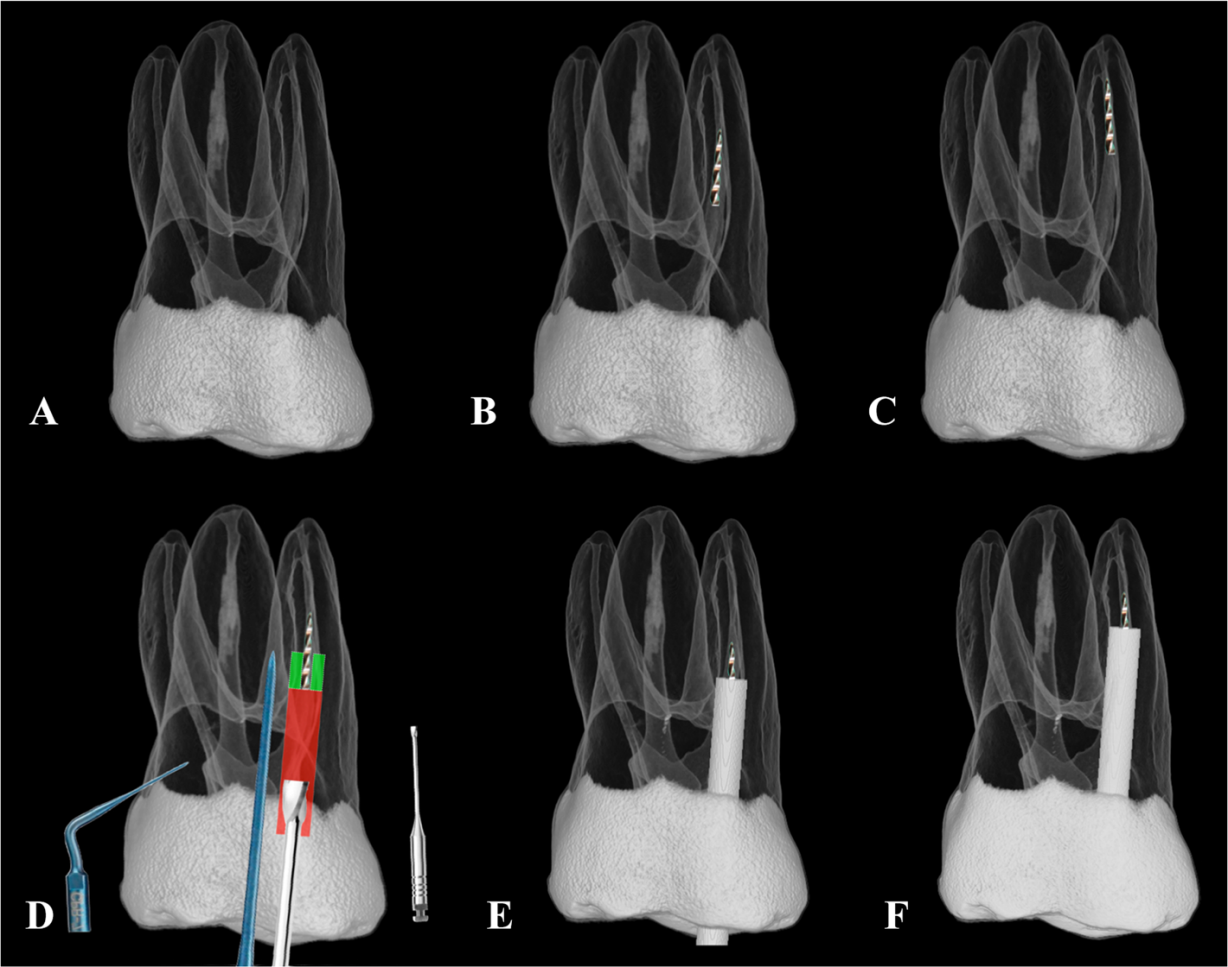
**a** Morphological reconstruction of one maxillary first molar; **b** & **c** size 25/.06 NiTi instrument with 3 mm apical segment assumed to be fractured in the mesiobuccal canal with 3 mm and 5 mm away from the canal orifice; **d** using modified Gates Glidden burs to create a staging platform and CPR ultrasonic tip to trephine dentin a 1.5 mm distance apically from the coronal part of the fragment around the fragment; **e** access to the fragment at 3 mm; **f** access to the fragment at 5 mm

**Fig. 2:**
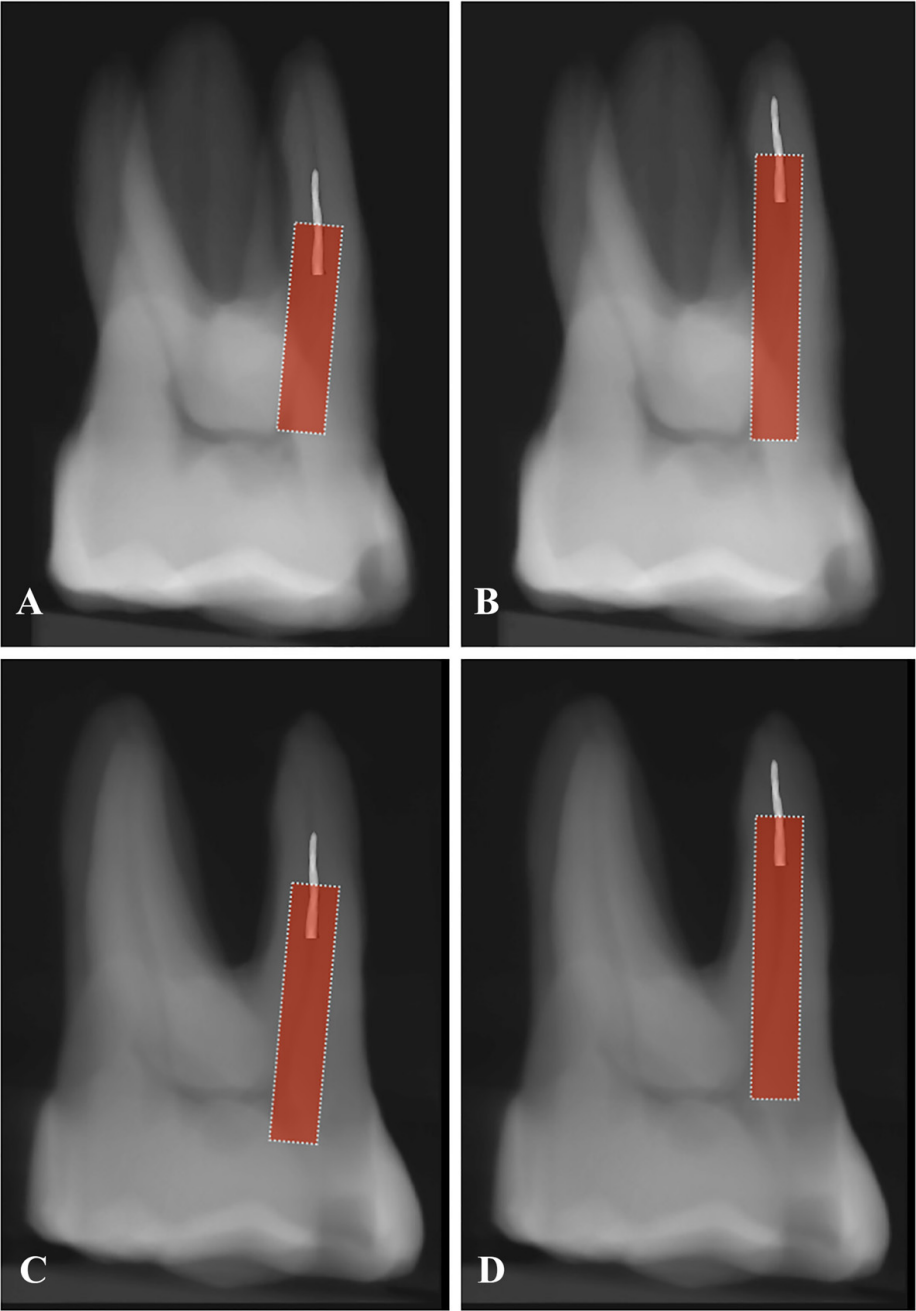
Simulated X-ray image by parallel and parallax technique when broken instrument below the orifice 3 mm (**a**, **c**) and 5 mm (**b**, **d**) and measurement by ImageJ software

The MeVisLab package (www.mevislab.de/index) (MeVis Medical Solution, Bremen, Germany) was used, which provided a visual data-flow program environment on a graphic user interface [19], to build a virtual simulation platform for the mesiobuccal (MB) canal of all specimens. The steps of the workflow were similar with those described in another study [19], and included the following steps: (i) Build a 3D dataset from the scanned maxillary molar image; (ii) a 3 mm-long apical segment of a size 25, taper 0.06 endodontic instrument was assumed to have fractured in the MB canal and situated at 3 or 5 mm below the orifice; this was created virtually in the 3D reconstructed model (Fig. 1b and c); (iii) the tooth model was rotated at various angles using the “DRR module” to “isolate” the mesiobuccal root by rotating the tooth model so that it was not overlapped by the palatal root; and (iv) simulated x-ray images, either paralleling or angled (parallax), were generated to represent radiographic images obtained clinically with the techniques, respectively (Fig. 2a-d).

### Virtual simulation of the removal of the broken fragment

The clinical procedures were simulated in the Mevislab package as follows: First, the tooth model created as in step (i) and (ii) above. Then, a modified Gates Glidden burs #4 was used to prepare, a “staging platform” up to the coronal aspect of the fractured piece; a scaled and dimensionally correct 3D image of the instrument was inserted into the model in software (see Fig. 1d). After that, ultrasonic tips, (CPR number 7, Obtura-Spartan, Fenton, MO, USA)were used to trephine the dentin around the fragment for 1.5 mm along the fragment (Fig. 1d) to allow the broken instrument to “jump out” of the canal or to retrieve it by using a micro-tube instrument removal system (Fig. 1). The most conservative space requirement was assumed in this simulated process: the diameter of the coronal end of the broken instrument (Db) was 0.43 mm for the 0.06 tapered file and the minimum diameter (D_c_) of the CPR ultrasonic at 0.4 mm. Therefore, theoretically, the diameter of the trough created by the ultrasonic tip (D = D_b_ + 2D_c_) was 1.23 mm. A cylindrical space of this diameter was positioned around the broken instrument uing the “SoTransformerDragger module” of MeVisLab (Fig. 1e and f).

The 2D simulation steps of fragment removal were performed in ImageJ software (http://imagej.nih.gov/ij/). First, simulated radiodgraphs were generated with a direct ray casting technique from the 3D dataset. Then, a rectangle (4.5 mm × 1.23 mm&6.5 mm × 1.23 mm) that corresponded to the space for straight-line access was set in the resultant paralleling and parallax x-ray images. A similar trepan space (1.23 mm diameter) was created by around the fragment (Fig. 2).

### Measurement of remaining canal wall thickness

**Fig. 3:**
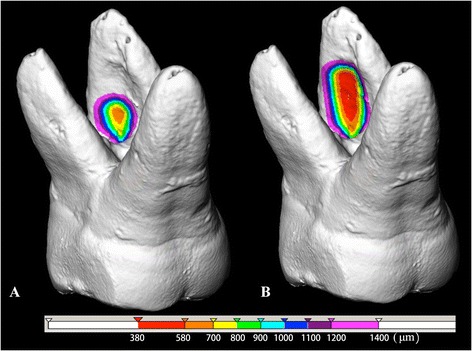
3D color-coded image of residual dentin thickness distribution around the narrow parallel space in root dentin after created a staging platform when the instrument placed in mesiobuccal canal below the orifice with 3 mm (**a**) and 5 mm (**b**) depth

Model dataset of each tooth after the simulation procedure was submitted to 3D measurement in Mevislab. The remaining dentin thickness measurements were made from the root canal wall to the external root surface along the root using the “3D SurfaceDistance module” of the software. These distances were stored in the nodes for color-coding and analysis. A 3D marker was placed on the surface to allow visualization of the dentin thickness there (Fig. 3). A 3D-RDT value was obtained for each tooth.

The 2D canal wall thickness was estimated on both the paralleling (Pa-RDT) and parallax radiograph in the ImageJ software. The RDT value was taken as the minimum distance from the side of the rectangle to the external root surface (Fig. 2).

### Statistical analysis

The RDT values were submitted to two-way analysis of variance. Then, the 3Dunnett t test was used to identify the differences in RDT between radiographic and actual 3D thickness. Intra-class correlation coefficient (ICC) was used to assess consistency between the radiographic and actual thicknesses. The level of significance was set at p < 0.05. All analyses were performed a statistical package (SPSS 21.0, SPSS Inc., Chicago, IL).

## Results

**Fig. 4:**
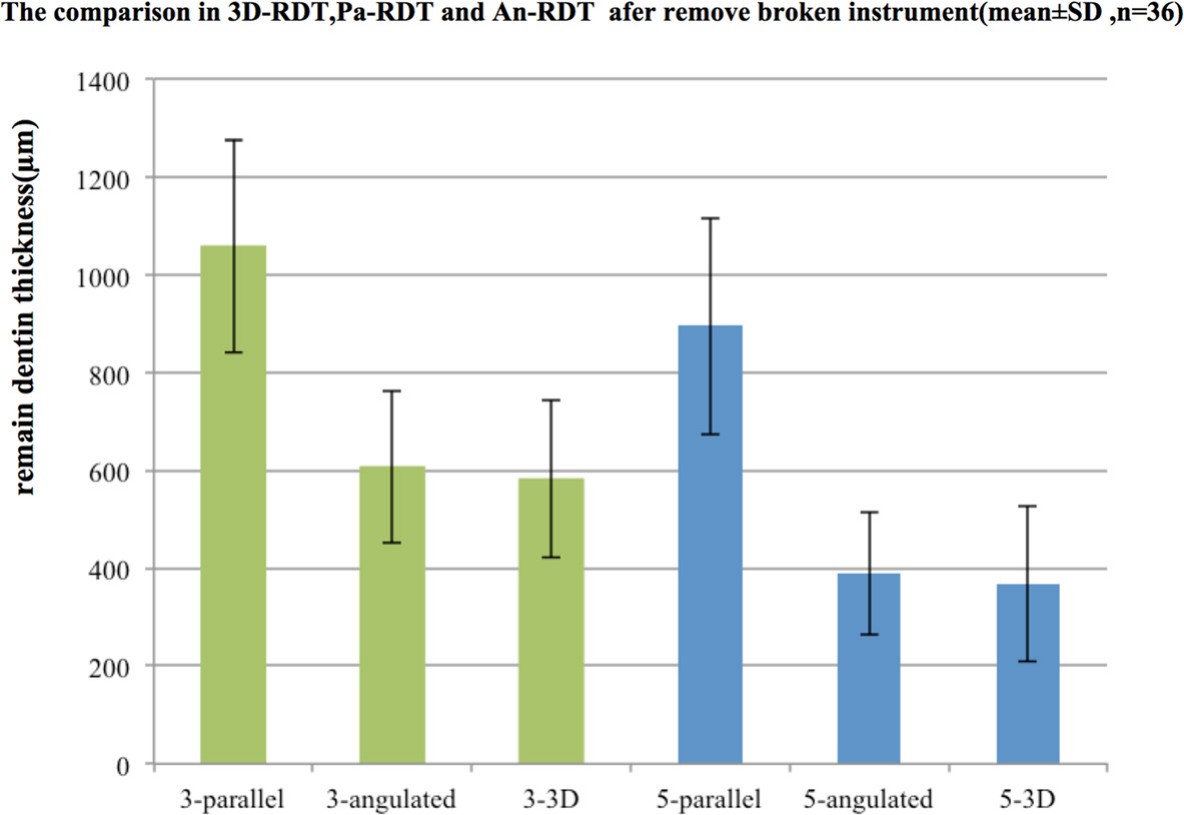
The means and standard deviations for RDT by different methods. 3D-RDT(=3D remain dentin thickness), Pa-RDT(= remain dentin thickness obtained from parallel technique), An-RDT(= remain dentin thickness obtained from angulated technique), green and blue color were instrument broken 3 mm and 5 mm below the orifice (group 3 mm and group 5 mm)

This virtual simulation platform can provide a safe environment for planning the removal of a broken instrument interactively. The often-proposed approach was followed, i.e. by creating a staging platform and then troughing around the fragment. The space created in such process was simulated in both the 2D and 3D datasets. RDT measurements were obtained from different radiographic projections and from the 3D analysis; the mean and standard deviations were deported in Fig. 4.

For the group with fragment 3 mm below orifice, the minimum RDT value obtained from paralleling radiographic technique (1058 ± 216 μm])was significantly greater than that by the parallax (angled) technique (An-RDT) (606 ± 155 μm), as well as the 3D-RDT (581 ± 159 μm) (p < 0.05). For the 5 mm-deep group, the An-RDT (389 ± 126 μm) was only slightly greater than 3D-RDT (368 ± 159 μm). The Pa-RDT was (895 ± 220 μm), which value was significantly greater than the former two (p < 0.05). Considering the effect of fragment location, the minimum RDT of the 3 mm-deep group was generally greater than that with fragments situated deeper (5 mm below the orifice) in the canal. There were no differences between parallax angled radiograph (An-RDT) and 3D-RDT value for both locations (3 mm versus 5 mm below the orifice) of the fragment. The ICC values of remaining dentin thickness measurements between the paralleling technique and the 3D analysis were 0.479 and 0.574 two for the fragment locations, respectively. Noted that both values were lower than 0.75. The ICC between parallax-RDT and 3D analysis were 0.721 and 0.667 for the two locations, which values were close to 0.75.

The average rotation angle from the paralleling technique to obtain a parallax radiograph with unimpeded image of the mesiobuccal root was 21.06 ± 4.34°.

## Discussion

In a recent survey conducted in the UK, 85.1 % of general dental practitioners and 94.8 % of endodontists have experienced fracture of endodontic instruments [20]. Instrument fracture often occurs in narrow and curved canals, such as the mesiobuccal canal of maxillary molars [21, 22]. Removing a fractured instrument from the root canal is a demanding task. Sufficient enlargement of the root canal coronal to the fragment is essential for successful retrieval. Usually a staging platform coronal to the fragment is prepared to allow straight-line access and direct sight of the fragment under the operating microscope. This is followed by the application of ultrasonic tips. If the direct application of ultrasonic energy does not loosen the fragment sufficiently to remove it, then there is a need to grab and retrieved the fragment with some variant of micro-tube [23].

Gao et al. [19] reported that the application framework, based on the freeware MeVisLab, enables the 3D reconstruction and measurements of root canal and teeth scanned by micro-CT. The virtual simulation platform can provide a safe environment for planning for the removal of fractured instruments. Virtual digital radiographs can be generated from the reconstructed micro-CT data. This permits an assessment of remaining dentin wall thickness, as estimated by plain radiographs, with the measurement from 3D analysis serving as the standard for comparison. The software platform in 3 dimensions has facilitated the realistic simulation and evaluation of any changes in dentin thickness that occurs in the roots, if the clinical procedure were to be performed. This platform also allows the comparison of dentin wall thickness obtained from radiographs taken different angles. The technique described in our present study allows each root to serve as its own control and overcomes the problem of sample variation. The virtual simulation platform provides useful and intuitive information in education and research, with potential to extend to the clinical situation.

During the removal of any broken instrument, dentin reduction must be done carefully to avoid root perforation. Hence, treatment planning should include a risk assessment. The clinician has to evaluate the options of either attempting to remove the fragment, bypassing it, or leaving the broken fragment inside the root canal. The decision is often based on information about the root canal wall thickness, especially when root fracture or perforation is to be avoided. The risk of the endodontically treated teeth to fracture increases proportionally to the amount of dentin removed [7]. A direct relationship exists between remain dentin thickness and the strength of the root [24–26]. Thus, preservation of sound dentin is very important during removal of broken instrument. In previous studies, teeth were sectioned at one or several selected levels of the root with measurements done in 2D in cross sections [11, 27]. Unavoidably, some parts of the root were destroyed during sectioning and could not be assessed. In the present study, all levels of the root were examined in a virtual platform that also permitted the quantification of the radicular wall thickness if an attempt was made for the broken instrument. The images may be color-coded for easy visualization of the result after these manipulations drilling and troughing were carried out in the tooth.

One may argue that cone beam computed tomography (CBCT) is an accurate and noninvasive technique that may be applied in the clinical situation. However, the cost and radiation dose to the patient must be considered. Periapical radiograph is likely to remain as the most important tool in clinical practice, which is a compromise when dentin thickness information is concerned. Raiden et al. [18] and Souza et al. [28] evaluated the post preparation in premolars using paralleling (bucco-lingual) radiographs, and concluded that periapical radiographs after overestimate the actual root canal wall thickness. Our present study supported the finding that paralleling radiographic technique would overestimate the actual RDT. On the other hand, the parallax technique seems give a closer or more accurate estimation of the actual RDT. As the root may display different appearance in varied projection angle, the projected shape and curvature of the mesiobuccal root could influence the measurement on a periapical radiograph. When the beam crosses the tooth at a certain angle (as in a paralleling technique), the tooth appears blurred in the radiograph. Thus, by the angulating the beam, the shape and concavity of the mesiobuccal root may be better visualized. This is reflected in the results that angled film (parallax technique) produced thickness measurement that is close to, but still slightly greater than the actual 3D-RDT. It might be related to the presence of concavities on the distal (or furcal) surface of the mesiobuccal root of maxillary first molars that were not visible radiographically and thus concealed the true distance between the outer root surface and the root canal wall. Simply put, plain radiographs provide an over-optimistic estimation of the dentin root canal wall thickness on the furcal aspect of the mesiobuccal root. Using a parallax technique would help to reduce the discrepancy in the thickness estimation for risk assessment.

For the actual RDT, the coefficient of variation was 0.034 and 0.049 in the two fragment-location groups (3 and 5 mm). When this coefficient was small, that ICC value would not be high [29]. The ICC values of RDT measurement between parallax radiograph and 3D analysis were close to 0.75 in the present study, suggesting that the parallax technique may provide a better prediction of the true thickness. The thicknesses estimated from these two methods were closer to each other, and were significantly different from that obtained from the paralleling radiographs. Thus, an angled radiograph should be taken when an attempt to remove the broken instrument from the MB canal of maxillary molar is contemplated.

Changing the angulation of the radiation source may help in determining the presence of root or strip perforation [30], additional roots, the localization of periradicular pathosis, and other anatomic structures. The parallax radiographs can avoid the problem of overlapping structures to some extent. For instance, the best angle would show the MB root clearly, separate from the distobuccal and palatal root. In the present study, this horizontal offset angle was about 21°. This may be a guide to the radiologist or clinicians when faced with a broken instrument in such a situation. Morphologically, the anatomy of the MB root of maxillary first molar was complex with a high incidence of MB2 canals, isthmuses, accessory canal, apical delta and loop [31]. Root canal curvatures are most pronounced in the MB canal, in which most cases of instrument fracture occur. In the coronal part, the furcal [i.e. distal] wall of the MB root is rather thin and, often, is much thinner than the mesial wall at similar level [32]. Realizing that intraoral radiographs will overestimate the RDT would be helpful for clinicians to make decisions during clinical procedures; the parallax technique is more accurate than paralleling technique in this regard.

## Conclusions

In conclusion, based on virtual simulation platform, the minimal remaining dentin thickness after attempt to remove a fracture instrument was affected by the projection angle, the position of the fractured instrument. There was a high risk of perforation in the middle third of the mesiobuccal canal in the maxillary first molar. Although the results from virtual simulation models cannot always completely extrapolate to the in vivo/patient situation, they can provide valuable insight into the benefit/risk analysis before removal of a separated instrument. To evaluate the RDT during remove broken instrument in maxillary first molars, parallel radiographs overestimate actual remain dentin thickness and angulated technique were significantly more accurate than parallel technique when the angle was 21°. It provides reference information for endodontists and radiologists.

## Abbreviations

RDT: Remain dentin thickness
MB: Mesiobuccal
3D: 3-dimensional
ICC: Intra-class correlation coefficient
NiTi: Nickel-titanium
Micro-CT: Micro-computed tomography
Pa-RDT: Remain dentin thickness obtained from parallel technique
An-RDT: Remain dentin thickness obtained from angulated technique
CBCT: Cone beam computed tomography
